# Red Yeast Rice and Optimal Fermentation Periods Improve the Quality of Esan Fermented Fish Sausage

**DOI:** 10.1155/2024/4831279

**Published:** 2024-03-27

**Authors:** Somsamorn Gawborisut, Suprawee Muengkratok

**Affiliations:** Fish Processing Laboratory, Department of Fisheries, Khon Kaen University, 123 Mittraphap Rd., Khon Kaen 40002, Thailand

## Abstract

Esan fermented fish sausage (EFFS) has an unappealing off-white color. The incorporation of an appropriate amount of red yeast rice (RYR) and the selection of an optimal fermentation period may yield visually appealing, high-quality sausages. This study is aimed at investigating the effects of different RYR levels (0, 0.35, and 0.7%) and fermentation periods (0, 2, 4, and 6 days) on the quality parameters of EFFS. The following parameters were examined for raw EFFS: CIE color values (L^∗^, a^∗^, and b^∗^), microbial analyses (total viable count, lactic acid bacteria, and yeast and mold counts), titratable acidity (TA), pH, weight loss, cooking loss, texture profile analysis (TPA), and sensory evaluation (color, odor, hand-feel texture, overall acceptability, and overall preference ranking). The quality parameters of the cooked EFFS were CIE color values and sensory evaluation (color, odor, mouthfeel, texture, flavor, overall acceptability, and overall preference ranking). The results showed that 0.35 and 0.7% RYR increased the a^∗^ (red/green) values of raw and cooked EFFS but decreased the L^∗^ (lightness) and b^∗^ (yellow/blue) values. These RYR levels significantly improved the sensory color, overall acceptability, and overall preference ranking of the raw and cooked EFFSs. However, no statistical differences were observed between the effects of 0.35 and 0.7% RYR. RYR levels did not affect the microbial analyses, TA, pH, weight loss, cooking loss, or TPA. Moreover, they had no effect on the odor and hand-feel texture of raw EFFS, or the odor, mouthfeel texture, or flavor of cooked EFFS. Therefore, RYR supplementation improved the color quality of the EFFSs without altering the other quality parameters, with 0.35% RYR deemed optimal. Moreover, the fermentation period significantly influenced most quality parameters, except CIE color values and sensory color perception of raw and cooked EFFSs. Most sensory parameters improved by day 2, remained unchanged until day 4, and then deteriorated on day 6.

## 1. Introduction

Fish is an excellent source of protein, easy to digest, and is thus perceived as a healthy food [1]. However, because fish are highly perishable [2], preservation methods are required to extend their shelf life. Fermentation is not only one of the most effective preservation methods for fish but also produces aroma compounds and a flavorful taste that enhances palatability for consumers [3]. Fermented fish sausages, one of the most popular fermented fish products worldwide, are typically produced from freshwater fish [4]. In Thailand, fermented fish sausage consists of minced fish, salt, cooked rice, and minced garlic wrapped in banana leaves or stuffed in plastic or natural hog casings. Subsequently, the sausage is allowed to ferment spontaneously at ambient temperatures (30°C) for 2–5 days until ripe [5]. Lactic acid bacteria (LAB) play an important role in sausage ripening by converting rice and other carbohydrates into organic acids, particularly lactic acid [6–8]. These acids produce a sour taste [6, 7] and decrease the pH, thus extending the shelf life and causing acid denaturation of fish muscle proteins [8]. Acid-denatured fish muscle proteins have a firm texture, thereby improving the mouthfeel of the product [8]. LAB proliferation during fish sausage fermentation produces various volatile compounds that contribute to the unique aroma of the product [9].

Thai fermented sausages typically consist of four basic ingredients: ground meat (beef, pork, or fish), salt, carbohydrates (cooked rice), and minced garlic. Two types of fermented fish sausages are produced in Thailand: regular fermented fish sausages (Som-fug or Nham Pla) and Esan fermented fish sausages (EFFSs; Sai Krok Esan Pla). Regular fermented fish sausages are composed of minced fish, salt, cooked rice, and minced garlic [7, 8], which are typically wrapped in banana leaves or stuffed in plastic casings. However, EFFSs contain additional ingredients, including minced fish, salt, cooked rice, minced garlic, sugar, pork backfat, glass noodles, and white pepper powder [10], which are stuffed into hog casings and tied into links.

EFFSs exhibit an unappealing off-white color compared to their pork or beef counterparts, which attain pink or red colors derived from added nitrite compounds. However, the use of nitrite compounds in all fish products, including EFFSs, is prohibited in Thailand due to the possibility of carcinogenic nitrosamine formation. To address this, an alternative natural food colorant, red yeast rice (RYR) or angkak, may be used in EFFSs to improve the color without altering the sausage fermentation process.

RYR is produced by fermenting white rice with *Monascus* spp. [11]. Although the molds can produce red (monascorubramine and rubropuntamine), orange (monascorubrin and rubropunctatin), and yellow (monascin and ankaflavin) pigments in rice [11], the red pigments are preferred. RYR, which expresses a red hue, has been used extensively in Asia for various purposes, including traditional medicine, food colorants, preservatives, and food supplements [11–13]. Therefore, food-grade RYR is considered a safe ingredient. Administration of 18 g/kg body weight of RYR did not lead to death or toxicity in mice [13]. Studies involving terrestrial animal sausages have demonstrated the efficacy of RYR in improving product color [14–20]. Furthermore, several researchers [13, 21–24] have explored the addition of RYR to fishery products to enhance color. However, the use of RYR to improve EFFS color has not yet been reported. Notably, Tirasarot and Wongtanon [25] found that RYR could enhance the color quality of emulsified fish sausages. However, the impact of RYR on EFFS quality during fermentation has not been reported. Therefore, this study is aimed at investigating the effects of varying RYR levels (0, 0.35, and 0.7%) and fermentation durations (0, 2, 4, and 6 days) on the quality parameters of EFFS.

## 2. Materials and Methods

### 2.1. RYR

RYR powder (Artchit International Pepper and Spice Co., Ltd., Bangkok, Thailand) was purchased from a local supermarket (Tesco Lotus, Khon Kaen, Thailand). It was used in the experiment by mixing it into minced fish used for EFFS preparation.

### 2.2. Minced Fish

Twelve kilograms of minced rohu (*Labeo rohita*) (Sompong's Minced Fish Meat, Ayutthaya Province, Thailand) was purchased between September and October 2022 from a local market, placed on ice, and transported to the Fish Processing Laboratory, Khon Kaen University, Thailand. The minced fish was mixed thoroughly and divided into three portions (4 kg/portion). These portions were randomly assigned three RYR levels (0, 0.35, and 0.7% minced fish). The selection of these three RYR levels was based on our preliminary investigation, which showed that RYR at 0.35% and 0.7% by weight of minced fish yielded EFFS with a^∗^ values closely resembling those of the top two most popular commercial fermented sausages. The 0% RYR served as the control. RYR was added to the fish and mixed for 3 min using a silent cutter (Cuttex M11; NMH, Maschinen, Germany). Subsequently, the fish was stored in plastic bags on ice for EFFS preparation.

### 2.3. EFFS Preparation

EFFS was prepared using a modified recipe from the Fisheries Industrial Technology Research and Development Division [10]. Four kilograms of minced fish was mixed with 140 g of salt for 3 min using a silent cutter. Subsequently, 500 g of garlic, 120 g of sugar, 2000 g of cooked rice, and 5 g of white pepper powder were combined and mixed with the fish for 4 min. Next, 1500 g pork backfat and 2000 g soaked glass noodles were added and briefly mixed for 40 s. The sausage mixture was stuffed into natural hog casings. Sterilized cotton strings were used to tie the sausages (5 cm length/sausage) into links. The prepared EFFSs were divided into four portions and placed into separate plastic bags. Four fermentation periods (0, 2, 4, and 6 days) were randomly assigned to these portions. These fermentation periods were selected based on our previous experiment indicating that EFFS, which was prepared from minced rohu, ripened on day 2, remained of good quality until day 4, and spoiled on day 6 (unpublished data). Subsequently, the four bags were placed in an incubator to ferment at 30 ± 1°C. EFFS samples were collected from each bag and subjected to quality analyses.

### 2.4. Quality Analyses

#### 2.4.1. CIE Color Values

The surface color of the raw and cooked EFFSs was measured using a Konica Minolta CM-2600d Spectrophotometer (Konica Minolta, Inc., Tokyo, Japan). A D65 artificial daylight bulb and a 10° standard-angle observer were used to illuminate the samples. Cooked sausages were prepared prior to color measurement by baking them in an oven at 200°C for 15 min until the internal temperature reached 70°C according to AOAC method 976.16 (35.1.04) [26]. Color values were expressed as L^∗^, a^∗^, and b^∗^ values, representing lightness, red/green, and yellow/blue coordinates, respectively.

#### 2.4.2. Microbial Analyses

Total viable count (TVC), LAB, and yeast and mold (YM) were determined using standard methods. Briefly, 25 g of EFFS was homogenized in 225 mL of sterilized 0.1% peptone water for 60 s using a 3500 Jumbo Stomacher (Seward Laboratory Systems Inc., Bohemia, NY, USA). Decimal dilutions (10^−1^ to 10^−7^) were prepared and 0.1 mL aliquots of each dilution were spread onto the appropriate media. TVC was determined using standard plate count agar (BBL, Sparks, MD, USA). The TVC plates were incubated aerobically at 30°C for 72 h [27]. LAB were measured using de Man, Rogosa, and Sharpe (MRS) agar (BBL, Sparks, MD, USA). Agar plates were incubated under anaerobic conditions at 30°C for 72 h [28]. YM were determined using potato dextrose agar (BBL, Sparks, MD, USA) acidified with 10% tartaric acid to a pH of 3.5. YM were enumerated aerobically at 22 ± 1°C for 5 days [29]. Colonies from all microbial analyses were counted, converted into logarithmic values, and expressed as log CFU/g.

#### 2.4.3. Titratable Acidity

The titratable acidity (TA), expressed as a percentage of lactic acid, was determined using the potentiometric titration method (AOAC 970.15B, 27.1.18B) [26]. Three grams of each EFFS sample was homogenized with 50 mL of recently boiled deionized water for 30 s. Subsequently, the homogenized samples were titrated with standardized 0.1 N NaOH until the pH reached 8.2. The volume of NaOH was recorded and used to calculate TA according to the equation (1) as follows:

(1) $$\begin{array}{c} \text{Titratable acidity }\left(\%\right)=\frac{V\times N\times 90\times 100}{g}\times 1000, \end{array}$$

where *V* is the volume (mL) of NaOH (0.1 N), *N* is the normality of NaOH, and *g* is the weight of the sample.

#### 2.4.4. pH

The pH of the raw EFFS was determined according to the method of Jung et al. [30]. Briefly, 10 g of EFFS was homogenized with 100 mL of recently boiled deionized water for 30 s. The pH of the homogenized samples was measured using a Sartorius PP150 pH meter (Sartorius Corp., Edgewood, NY, USA).

#### 2.4.5. Weight Loss

Weight loss was determined by recording the initial mass of the EFFSs from each treatment at the beginning of the experiment and the final mass at the end of each fermentation period. The difference between the initial and final masses was calculated and expressed as a percentage of the initial mass.

#### 2.4.6. Cooking Loss

Cooking loss was calculated similarly, with the difference before and after cooking. The EFFS mass was recorded before cooking. Subsequently, the sausages were baked in an oven at 200°C for 15 min until the internal temperature reached 70°C, as previously described. The cooked sausages were then strained to drain the cooking liquid, and the mass of the cooked sausage was recorded. The difference in the mass of EFFS before and after cooking was calculated and expressed as a percentage of the mass before cooking.

#### 2.4.7. Texture Profile Analysis (TPA)

TPA was performed using a texture analyzer (TA-XT2i, Stable Micro Systems Ltd., Vienna, Austria) equipped with a 75 mm platen. The EFFSs were cut into cylindrical shapes (height: 25 mm), measured at a crosshead speed of 60 mm/min, and compressed twice to 40% of their original height [31]. Six cylindrical samples obtained from each treatment were subjected to TPA. The following TPA parameters were reported: hardness (g), adhesiveness (g.sec), springiness, cohesiveness, and chewiness (g).

#### 2.4.8. Sensory Evaluation

The sensory acceptability and overall preference rankings of raw and cooked EFFSs were evaluated separately by panelists accustomed to fermented fish sausages. Prior to all sensory evaluations, panelists were informed about the composition of the EFFS to ensure that they were able to withstand the fish smell and were not allergic to the sausage. Raw EFFS samples were evaluated for sensory acceptability (color, odor, hand-feel texture, and overall acceptability) and overall preference rankings by 45 panelists. The panel comprised 25 females and 20 males, ages 19–45 years. Cooked EFFS was prepared by baking raw sausages in an oven at 200°C for 15 min until the internal temperature reached 70°C, as previously described. Subsequently, it was cooled to ambient temperature for 20 min and evaluated for sensory acceptability scores (color, odor, mouthfeel texture, flavor, and overall acceptability) and overall preference rankings by 45 panelists. The panel comprised 23 females and 22 males, aged 19–45 years. The sensory acceptability of raw and cooked EFFSs was determined using a nine-point hedonic scale as described by Meilgaard et al. [32] (1 = dislike extremely, 5 = neither like nor dislike, and 9 = like extremely). Prior to each evaluation, the samples were randomly assigned a three-digit number and presented to the panelists. A score of five was considered the cutoff point for all sensory acceptability attributes. The overall preference ranking was determined based on the method by Lu [33]. A ranking of “1” indicated the most preferred. All sensory evaluations were conducted in an air-conditioned room at 25°C. Illuminance levels of 503-512 lx on the sensory tables were arranged.

### 2.5. Statistical Analysis

Data were analyzed using the univariate procedure in the IBM SPSS Statistics 21 program (IBM, Armonk, NY, USA) to verify data distribution and assess the normality and homogeneity of variance. A 3 × 4 split-plot arrangement within a randomized complete block design (RCBD) was employed. The main plot was the RYR level (0, 0.35, and 0.7% minced fish), and the subplot was the fermentation period (0, 2, 4, and 6 days). The experiment was performed in triplicates using three blocks of RYR produced from three different processing lots. The effects of the RYR level and fermentation period on the quality parameters of EFFSs were analyzed using the IBM SPSS Statistics 21 program at a 95% confidence level. Mean values were compared using the least significant difference (LSD) test.

## 3. Results and Discussion

The results revealed no significant interactions between RYR levels and fermentation periods for any quality parameter (*p* > 0.05). Therefore, for each quality parameter, the RYR levels and fermentation periods are presented and discussed separately. Statistical analyses showed that the three blocks of the RYR were not significantly different (*p* > 0.05).

### 3.1. CIE Color Values

**Figure 1:**
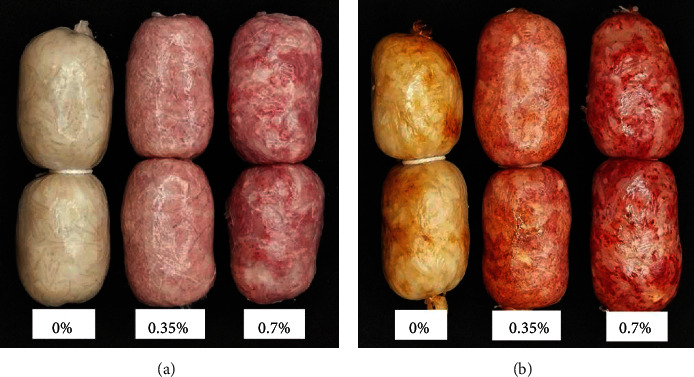
Effect of the level of red yeast rice on the color of (a) raw and (b) cooked Esan fermented fish sausage.

EFFS is typically purchased raw. Therefore, raw EFFS with an appealing color may influence consumers' purchasing decisions. However, the color of cooked EFFS may affect consumers' perceptions prior to consumption and the overall eating experience of the sausages. The results indicate that increasing RYR levels significantly decreased the L^∗^ and b^∗^ values of raw and cooked EFFS (*p* < 0.05) (Table 1). However, increasing the RYR level significantly increased the a^∗^ value (*p* < 0.05). The enhanced red color of raw and cooked EFFS is shown in Figure 1. These results indicate that RYR improves the redness value of EFFS, and the redness did not fade after cooking.

The RYR used in the experiment exhibited a deep red color with L^∗^, a^∗^, and b^∗^ values of 28.66, 22.18, and 9.60, respectively. This intense red color (high a^∗^ value) may be attributed to the red pigments in the RYR, such as monascorubramine and rubropuntamine. The minced fish used in this experiment had a pale orange color, with L^∗^, a^∗^, and b^∗^ values of 49.16, 3.93, and 9.38, respectively. After adding RYR to the fish, its redness increased, consequently enhancing that of the EFFS. Therefore, the improved redness of the EFFS may be attributed solely to the addition of RYR. Chanshotikul and Hemung [34] noted that the redness of Thai fermented sausages must be maintained or improved by adding nitrite compounds or other alternative colorants, underscoring the significance of redness as a quality parameter for sausages. In summary, RYR addition improves the a^∗^ values, thereby improving the color quality of EFFS.

Abdollahi et al. [35] reported that *Monascus* pigments in RYR were moderately stable when exposed to low pH or high temperatures. However, our experiment demonstrated that the red color of EFFS persisted throughout the fermentation process at low pH and cooking at high temperatures. Therefore, RYR may be suitable as an EFFS supplement. Despite the absence of previous studies on the use of RYR in fermented fish sausages, our results align with the findings of Oksuz et al. [29], who investigated RYR supplementation in emulsified fish sausage. They observed a decrease in L^∗^ and b^∗^ values with increasing RYR levels, while the a^∗^ values of the sausages increased.

The experiment revealed that the fermentation period did not significantly affect the color values of the EFFSs (*p* > 0.05). Although protein denaturation and exudate loss, which occur during the fermentation of fish sausages [36], may impact the color values, no significant changes were observed in EFFS color values during the 6-day fermentation period. Specifically, L^∗^ values ranged from 54.28 to 56.16, while a^∗^ and b^∗^ values ranged from 10.41 to 11.17 and 8.64 to 9.14, respectively, during fermentation. After cooking, the EFFSs exhibited L^∗^, a^∗^, and b^∗^ values ranging from 47.53 to 49.01, 9.16 to 12.12, and 9.64 to 10.30, respectively. These results indicate that the fermentation period did not alter the color of the EFFSs, including redness in the sausages originating from RYR. These findings suggest that the color quality of EFFS can be maintained if the product is purchased and consumed within 6 days of production.

### 3.2. Microbial Analyses

TVC, LAB, and YM counts were not significantly affected by RYR levels (*p* > 0.05) (Table 1). This insignificance may be attributed to the low levels of RYR used in the experiment. This finding implies that RYR supplementation did not alter the fermentation pattern of EFFS. Although RYR was inoculated with *Monascus* spp. [11], the mold did not increase the YM count in the EFFS. The RYR used in the experiment may have been subjected to a pasteurization process prior to selling, potentially containing negligible amounts of YM.

**Figure 2:**
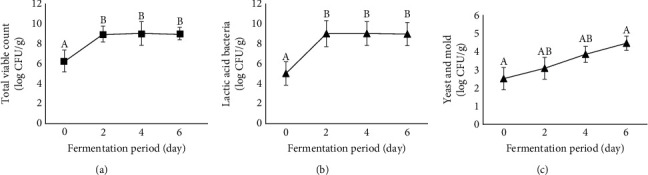
Effect of the fermentation period on the total viable count, lactic acid bacteria, and yeast and mold in Esan fermented fish sausage. Letters (A, B) within each quality parameter indicate a significant difference (*p* < 0.05).

However, the fermentation period significantly influenced the TVC, LAB, and YM counts (*p* < 0.05) (Figure 2). At the beginning of the experiment (day 0), TVC and LAB levels were 6.33 log CFU/g and 5.03 log CFU/g, respectively. The bacterial counts increased rapidly by day 2, reaching levels of 8.98–9.02 log CFU/g, and remained stable thereafter. These results align with those of Sangjindavong et al. [37], who observed stable TVC and LAB levels in fermented barracuda during days 2–7 of fermentation.

**Figure 3:**
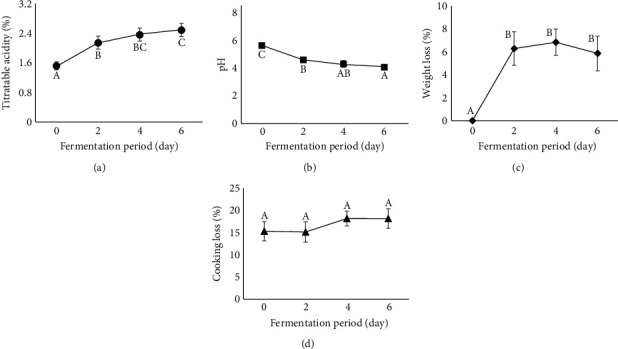
The impact of the fermentation period on titratable acidity, pH, weight loss, and cooking loss. Letters (A–C) within each quality parameter indicate a significant difference (*p* < 0.05).

LAB play key roles in fish sausage fermentation [3, 37, 38]. Several LAB species produce amylase, which converts starch into glucose [39]. Subsequently, glucose is metabolized into lactic acid by LAB via glycolysis [39]. Generally, LAB produce significant amounts of secondary metabolites such as organic acids, particularly lactic acid, during the stationary phase [40]. The accumulation of acid and volatile compounds produced by LAB enhances the aroma, flavor, texture, and shelf life of fermented fish products, including fermented fish sausages [6, 8, 36, 39]. Sangjindavong et al. [37] demonstrated that the stationary phase of LAB may begin on day 2 and continue throughout the fermentation process. The findings from the present study indicate that a significant amount of lactic acid may be produced from day 2 onwards, leading to increased TA and decreased pH in the EFFS (Figures 3(a) and 3(b)). Therefore, reaching the LAB stationary phase on day 2 may significantly improve the quality of EFFS.

YM counts in the experimental EFFS increased from 2.51 log CFU/g on day 0 to 4.46 log CFU/g on day 6. Similarly, Nie et al. [38] reported an increase in YM content during fish sausage fermentation. Yilmaz and Berik [41] showed that the ripening of trout-fermented sausage occurred on day 7 when YM reached 4.23 log CFU/g. Notably, the ripening duration of fermented fish sausages may vary depending on intrinsic and extrinsic factors such as microbial loads and communities, fermentation temperature, and nutrients supporting microbial growth. Therefore, the YM counts of each fermented fish sausage may differ during the ripening period. Although YM levels in fermented fish sausages have been reported, the presence of microorganisms may not necessarily be related to the ripening process. However, YM may potentially contribute to product spoilage if present in excessive amounts.

Wongwipairojkul and Siripornkitti [42] stated that YM in low-salt Thai fermented fish products, including fermented fish sausage, are mostly acid tolerant and potentially spoil the products. Although acidity levels (referred to as titratable acidity) increased during the extended fermentation period (Figure 3(a)), YM could withstand these conditions and consequently proliferate during fermentation, possibly causing spoilage of the products. Punyauppa-path et al. [43] reported that *Candida* spp. were the second most dominant molds in low-salt Thai fermented fish (Pla-Som), highlighting their potential role in product spoilage. Although the Thai Industrial Standards Institute [44] has not established specific YM standards for EFFS, the increasing amount of YM during the fermentation process in this study, coupled with findings from literature, indicate that YM may contribute to the spoilage of fermented fish sausages.

### 3.3. Titratable Acidity

The results showed that the TA was not significantly affected by the RYR levels (*p* < 0.05) (Table 1). Belleggia et al. [3] and Nooniam [36] reported that acids, mainly lactic acid, in fermented fish sausages are converted from rice during fermentation by LAB. The LAB results demonstrate that different levels of RYR yielded statistically similar LAB counts (Table 1). Therefore, similar levels of LAB may yield similar levels of acid, which may consequently produce similar TA levels.

**Figure 4:**
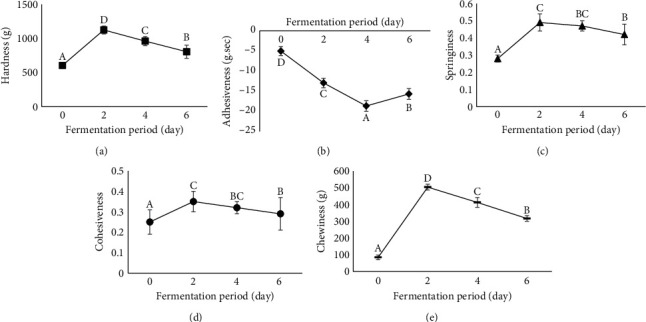
Effect of the fermentation period on the (a) hardness, (b) adhesiveness, (c) springiness, (d) cohesiveness, and (e) chewiness of Esan fermented fish sausage. Letters (A–D) within each quality parameter indicate a significant difference (*p* < 0.05).

**Figure 5:**
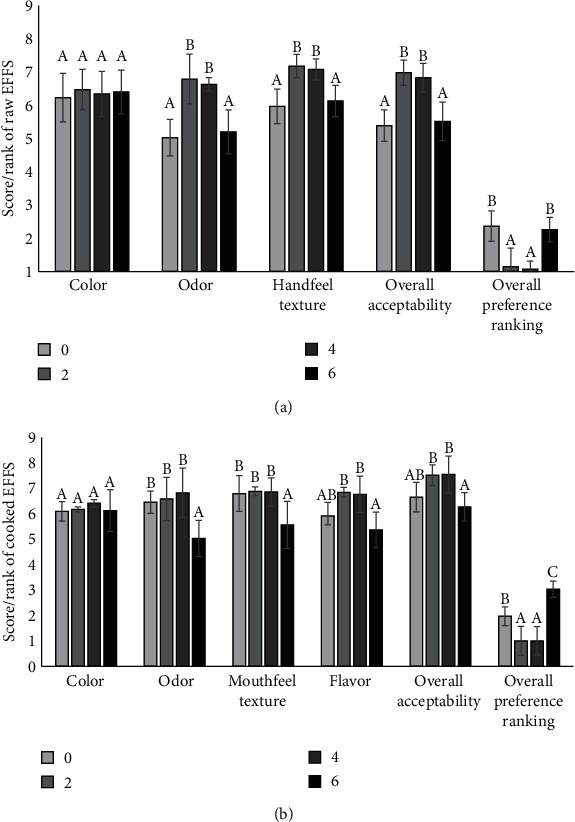
Effect of fermentation period on quality attributes of (a) raw and (b) cooked Esan fermented fish sausage. Letters (A–C) within each quality attribute indicate a significant difference (*p* < 0.05).

TA was significantly affected by the fermentation period (*p* < 0.05) (Figure 3(a)). The results showed that the TA increased as fermentation progressed. The TA in the experiment may reflect the amount of lactic acid converted from carbohydrate sources by LAB. An et al. [45] reported that increased TA in fermented fish products is related to LAB growth. A significant increase in TA was observed on day 2 when the LAB reached the stationary phase. Lactic acid, presumably the main secondary product, may be produced at the beginning of the stationary phase on day 2, thereby significantly increasing TA. The acids, particularly lactic acid, generated on day 2 were not only directly related to the quality of EFFS but may also reduce pH (Figure 3(b)). This reduced pH may further denature fish muscle proteins, consequently improving the TPA values (Figures 4(a) and 4(c)–4(e)) and the hand-feel texture of the product (Figure 5(a)) on day 2.

These results indicate that the TA gradually increased as the fermentation period increased. LAB in EFFS may continuously produce lactic acid during the extended fermentation period, thus increasing TA levels. These results align with those of Nooniam [36] and Xu et al. [46], who observed an increase in the TA in fermented fish sausages as fermentation progressed. The value of TA can be used to determine the acceptability of fermented fish sausages. Riebroy et al. [6] stated that TA levels of 2.2%–2.5% yielded the most acceptable fermented fish sausage. On day 2, the EFFS exhibited a TA of 2.14%, which was close to this range. Therefore, sausages may be acceptable on day 2. Similarly, on day 4, the EFFS had a TA of 2.37%, which was within the acceptable range. However, on day 6, the TA was 2.49%, nearing the upper limit of 2.5%. Thus, the sausage may have been unacceptable on day 6. These results indicate that EFFS remained acceptable for a short period from days 2 to 4 and became unacceptable on day 6. Although the fermentation period improved the quality of the EFFS, the window of improvement was limited to days 2 to 4, with potential deterioration evident by day 6.

### 3.4. pH

RYR levels did not significantly affect the pH of the EFFSs (*p* > 0.05) (Table 1). These findings suggest that the addition of RYR did not change the fermentation pattern of the EFFS. However, the fermentation period significantly affected the pH (*p* < 0.05) (Figure 3(b)). The results showed that the pH decreased as the fermentation period increased. An et al. [45] stated that the pH reduction in fermented fish products, including EFFSs, is directly related to LAB growth. LAB play a crucial role in converting carbohydrates in fermented fish sausages into acids, particularly lactic acid [3, 9, 36]. Although fish meat has a buffering capacity [47], the significant amount of acid produced by LAB during the extended fermentation period may have gradually decreased the pH of the EFFS. The pH values were strongly correlated with LAB growth and TA. LAB growth, particularly at the beginning of the stationary phase on day 2, significantly increased the TA and consequently reduced the pH in the EFFS.

The reduction in the pH of the EFFS during fermentation aligns with that in previous studies [7, 46, 48, 49]. According to the Thai Industrial Standards Institute [44], ripe fermented fish sausages ready for consumption must have a pH of ≤4.6. The low pH measurement of 4.58 detected on day 2 indicated that the sausages were ripe. Therefore, a fermentation period of 2 days was sufficient. In Thai and Chinese fermented fish sausages, pH reduction to the desirable level of ≤4.6 on day 2 was also reported by Wongsripaisan [7] and Xu et al. [46]. However, in Indonesian fermented fish sausages, Afifah et al. [48] and Nursyam et al. [49] observed pH values of >5 and ~4.5 on days 3 and 14, respectively. This variation in pH reduction in fermented fish sausages may be attributed to differences in LAB levels. Belleggia et al. [3] highlighted that LAB in fermented sausages originate from the raw materials used in sausage production and/or processing environment. The sausages used in the present study and those reported in the literature may contain different raw materials and be subjected to distinct processing environments. As a result, they could harbor different LAB loads and compositions, leading to varying pH reduction patterns.

### 3.5. Weight Loss

The RYR levels were not significantly different in terms of weight loss (*p* > 0.05) (Table 1), indicating that RYR supplementation did not adversely affect EFFS weight. However, the fermentation period significantly affected weight loss in the sausages (*p* < 0.05) (Figure 3(c)), with a significant increase observed on day 2 that remained consistent thereafter. This pronounced increase in weight loss on day 2 may be attributed to changes in the nature of the proteins in the EFFSs due to pH reduction. Sorapukdee et al. [31] reported that the extent of drip loss during fermentation is mainly dependent on the capacity of meat proteins to retain water. As the pH of the product approaches the isoelectric point (pI) of major proteins (especially myosin, pI = 5.4), the net charge of the protein becomes zero, reducing water attraction to the proteins and resulting in high drip loss during fermentation. Additionally, Riebroy et al. [8] stated that the water-holding capacity of myofibrillar proteins is minimal at pH < 5.0, further contributing to drip loss from the meat. A pH of 4.58 detected in EFFS on day 2 may have decreased the water-holding capacity of myofibrillar proteins in fish meat, consequently leading to significant weight loss in the sausage. Notably, the effect of fermentation duration on weight loss in fermented fish sausages has not been documented. In fermented mutton sausage, Cruxen et al. [50] observed an increase in weight loss as the fermentation period increased. Conversely, Sorapukdee et al. [51] observed a decrease in weight loss of fermented pork sausages decreased as the fermentation period increased.

### 3.6. Cooking Loss

The RYR levels had no significant effect on cooking loss (*p* > 0.05) (Table 1). Moreover, the cooking loss of the EFFSs was not significantly affected by the fermentation period (*p* > 0.05) (Figure 3(d)). Ezegbe et al. [52] highlighted that cooking loss should ideally be below 10% in fresh sausages and below 15% in fermented (semidry and dry) sausages. However, we observed cooking losses ranging from 15.11 to 18.14% in the EFFSs, which exceeds the acceptable value of 10%. To mitigate such losses and enhance product quality, exploring methods to retain moisture in EFFSs, such as incorporating natural dietary fibers, warrants further investigation.

### 3.7. TPA

The RYR levels did not significantly influence the TPA values of the EFFS (*p* > 0.05) (Table 1), indicating that the addition of RYR did not adversely affect the instrumental texture of the sausages. However, the fermentation period significantly affected the TPA values (*p* < 0.05) (Figures 4(a)–4(e)). Hardness, springiness, cohesiveness, and chewiness were low on day 0, with improvements observed on day 2 and gradually declines thereafter. Conversely, adhesiveness improved continuously from days 0 to 4, with a slight decrease observed on day 6. Overall, the texture of the EFFSs improved on day 2, followed by a gradual deterioration over subsequent days.

Riebroy et al. [53] reported that the hardness of fermented fish sausages is indicative of the degree of maturation. An increase in hardness results from the denaturation and gelation of meat proteins and water loss. Texture development in fermented fish sausages is closely associated with fermentation, during which meat binding is initiated by acid-mediated reactions. The decrease in pH gradually induces the aggregation of proteins, leading to the ordered formation of a protein structure, which is associated with firmness. The significant increase in the TPA values of the EFFSs on day 2 may be attributed to the significant reduction in pH detected on that day (Figure 3(b)), indicating sausage maturation. This pH reduction may have caused the ordered formation of the protein structure, thereby enhancing the instrumental texture values. A similar increase in the hardness of Indonesian fermented fish sausages on day 2 was reported by Afifah et al. [48].

The gradual reduction in TPA values after day 2 may have been caused by proteolytic enzymes originating from fish and those produced by LAB. Lougovois and Kyrana [54] highlighted the effectiveness of cathepsin D, the main fish muscle enzyme responsible for the hydrolysis of fish meat, at a pH of approximately 4. The pH reduction in EFFS to levels of 4.09–4.28 on days 4–6 may facilitate the activity of this enzyme, enhancing meet structure hydrolysis and reducing TPA values after day 2. Additionally, Cao et al. [55] reported the presence of LAB capable of producing proteolytic enzymes in fermented sausages. Proteolytic enzymes produced by LAB may further contribute to meat degradation and subsequent reductions in TPA values after day 2.

### 3.8. Sensory Evaluation

RYR levels significantly affected the color scores, overall acceptability scores, and overall preference rankings of raw and cooked EFFSs (*p* < 0.05) (Table 2). However, they had no significant effect on the odor and hand-feel texture scores of raw EFFS, or on the odor, mouthfeel texture, or flavor of cooked EFFS (*p* > 0.05). Specifically, 0.35% and 0.7% RYR yielded significantly higher color scores than 0% RYR (*p* < 0.05). However, there were no significant differences in color scores, overall acceptability scores, or overall preference ranking between 0.35% and 0.7% RYR (*p* > 0.05). This suggests that using RYR as high as 0.7% did not produce better results than using 0.35%, indicating that 0.35% RYR was sufficient to enhance the sensory qualities of raw and cooked EFFS.

Color is a crucial factor in consumer food choices [48]. Panelists preferred EFFSs exhibiting a red hue, characteristic of RYR supplementation (high a^∗^ values), over those with an off-white color. Moreover, RYR supplementation led to improvements in overall acceptability scores and preference rankings for EFFS. The RYR levels added to meat products may vary depending on consumer color expectations. For example, Tirasarot and Wongtanon [25] concluded that adding 1.2% RYR to fish meat was the most suitable level for enhancing the color quality of the emulsified fish sausage. In corned fish, RYR supplementation with 2.5% fish meat is the most suitable [56]. The most preferred Thai fermented pork sausage contains 0.75% RYR [57]. The findings from the present study indicate that 0.35% RYR supplementation is sufficient to meet consumer expectations for EFFS color. Notably, this supplementation produced raw EFFS with a^∗^ value ranging from 14 to 15 (Table 1). Therefore, the desired color for EFFS should have an a^∗^ value of approximately 14–15. Understanding this expected range of a^∗^ values may aid EFFS production when using different RYR sources. Regardless of the source, RYR levels capable of producing a^∗^ values within the ranges of 14–15 may yield a desirable EFFS color.

The fermentation period significantly affected the odor score, hand-feel texture score, overall acceptability score, and overall preference ranking of raw EFFS (*p* < 0.05) (Figure 5(a)). The odor scores of raw EFFS on days 0 and 6 were significantly lower than those on days 2 and 4 (*p* < 0.05). The panelists' records revealed that the lower odor score in the EFFS on day 0 was caused by the absence of a fermentation odor and the presence of a fishy smell. Odor scores significantly improved from day 2 to day 4. This improvement was due to the desirable fermentation odor that developed during this period. These findings suggest that EFFS may reach ripeness by day 2, with the desirable odor persisting from days 2 to 4. Subsequently, a significantly lower odor score of 5.19, which was close to the cutoff score, was detected on day 6. This low score was caused by the unpleasant pungent odor of the sample.

Zhao et al. [9] reported that most odorants in tilapia-fermented sausages are produced by microorganisms, mainly LAB, and by chemical reactions. These odorants contained 30–32 aldehydes, 14 alcohols, 13 hydrocarbons, nine ketones, five furans, three sulfur compounds, three aromatic compounds, three esters, two sulphurs, one nitrogenous compound, and one organic acid [4, 9]. High levels of these odorants may gradually accumulate in the EFFS matrix, resulting in a low odor score and shelf life termination by day 6. Similarly, the results of cooked EEFS also showed a low odor score (5.03) on day 6 (Figure 5(b)). This score was close to the cutoff score of 5, thus confirming that the shelf life of EFFS may terminate by day 6. Based on the odor scores, EFFSs require 2 days at room temperature to ripen and should be consumed on days 2–4 of the ripening period. The shelf life of EEFS may cease on day 6 because of its overripe pungent odor. These results align with those of Wongsripaisan [7] and Belleggia and Osimani [58], who reported a ripening period of 2 days for Thai fermented fish sausages. However, the shelf life of 6 days in our experiment with fermented fish sausage differs from that of approximately 5 days under ambient conditions reported by Belleggia and Osimani [58] and Valyasevi and Rolle [59]. The disparity may be attributed to differences in the loads and communities of LAB originating from the raw materials and processing environments.

The texture scores of raw EFFSs were significantly influenced by the fermentation period (*p* < 0.05) (Figure 5(a)). On day 0, the hand-feel texture score was significantly low. Subsequently, the scores improved on days 2–4 and declined on day 6. The panelist records revealed that the soft texture, which is commonly found in unripe fermented fish sausages, caused a low texture score on day 0. The texture score improvement observed on days 2–4 was due to a desirable firmer texture likely resulting from protein aggregation occurring at the low pH on day 2. The reduction in texture score on day 6 was caused by an undesirable mushy texture, potentially caused by degradation by proteolytic enzyme activity originating from fish tissues and LAB. Notably, the panelists' hand-feel texture scores seemed to correlate with the TPA values obtained from the texture analyzer. The mouthfeel texture scores of cooked EFFS were unchanged from days 0 to 4 (Figure 5(b)). However, it decreased by day 6, attributed to a noticeable soft texture perceived by the panelists. This undesirable soft texture likely resulted from degraded fish owing to proteolytic enzyme activity in raw EFFSs on day 6. These results imply that the shelf life of EFFS may be terminated on day 6.

The flavor scores of cooked EFFSs were significantly affected by the fermentation period (*p* < 0.05) (Figure 5(b)). Flavor scores were low at the beginning of the experiment. Subsequently, the scores increased on days 2–4 and decreased on day 6. The panelists' records revealed that the EFFS sampled on day 0 had an undesirable fishy smell during chewing. Additionally, it lacked sourness and a desirable fermentation flavor and thus received a low score. The scores improved on days 2–4 because of the proper levels of sourness and perceived fermentation flavor. These levels of sourness and flavor may originate from suitable levels of acids and flavor components produced by LAB during the ripening period. The declining score on day 6 was caused by the high level of sourness and unpleasant flavor detected during chewing. The flavor score of 5.36 rated on day 6 was close to the cutoff score of 5. Excess amounts of acids and flavor components produced by LAB may accumulate in the EFFS during the extended fermentation period and subsequently cause a significant reduction in the flavor score on day 6. This result indicates that the EFFS expired on day 6.

The overall acceptability scores and preference rankings of raw and cooked EFFSs were affected by the fermentation period (*p* < 0.05) (Figures 5(a) and 5(b)). The results showed that the scores and rankings were low at the beginning of the experiment on day 0. Subsequently, they improved on days 2–4 and decreased on day 6. These results align with other sensory attributes, showing that the improvement in the quality of the EFFS was mostly observed on days 2–4, and deterioration was generally observed on day 6. In summary, sensory scores and overall preference rankings indicate that the EFFSs matured on day 2, thus requiring 2 days to ripen. The quality of the EFFSs improved on days 2–4, suitable for consumption during this period. The quality of the sausage deteriorated on day 6. Therefore, the shelf life of EFFS is approximately 6 days under ambient conditions. To extend the shelf life of the product, matured EFFSs should be refrigerated.

## 4. Conclusions

Supplementation of RYR at levels of 0.35% and 0.7% improved the CIE color values, sensory color scores, overall acceptability scores, and overall preference rankings of the EFFSs. However, there were no significant differences between the effects of 0.35% and 0.7% RYR, indicating that 0.35% RYR was sufficient to improve the color of the product. RYR supplementation did not adversely affect microbial content, TA, pH, weight loss, cooking loss, or TPA values. Additionally, it did not alter the odor and hand-feel texture scores of the raw EFFSs, or the odor, mouthfeel texture, or flavor of the cooked EFFSs. These results indicate that RYR supplementation can improve the color quality of EFFSs without altering other quality parameters or fermentation duration.

The fermentation period significantly influenced most of the quality parameters of the EFFS, except for the CIE color values and sensory color scores. Most quality parameters did not meet the requirements at the beginning of the experiment (day 0 of fermentation). However, improvements were observed by day 2, indicating that the EFFSs achieved maturity and readiness for consumption by this time. Most quality parameters were maintained for a short period, from days 2 to 4 but declined on day 6. Thus, the shelf life of EFFSs should be terminated on day 6. The extension of the shelf life of a product using refrigeration should be explored further. Additionally, the high cooking loss observed in EFFS highlights the potential benefit of reducing losses through the addition of food additives or ingredients such as dietary fibers, which warrants further investigation.

Although nitrate or nitrite is permitted as a food additive in some countries for certain fishery products, their use should be avoided because of the potential formation of carcinogenic nitrosamines. This study demonstrates the possibility of improving the attractive color of fermented fish products using a safe natural colorant. Using RYR for red color enhancement in fermented fish sausages is recommended in Thailand and other locations where sausages are popular, nitrate or nitrite compounds are prohibited, RYR is available, and red color in the product is desired. The results of this study may inspire researchers globally interested in nitrate/nitrite-free fishery products to explore available local colorants to enhance the color of these products.

## Figures and Tables

**Table 1:** Effect of the level of RYR on quality parameters of raw and cooked Esan fermented fish.

Quality parameters	Level of RYR (% of minced fish meat)		
	0	0.35	0.70
Color value of raw EFFS			
L^∗^	64.63 ± 1.49c	53.03 ± 1.70b	48.10 ± 1.32a
a^∗^	1.38 ± 0.52a	14.25 ± 1.27b	17.00 ± 0.65c
b^∗^	11.17 ± 0.75c	8.15 ± 0.45b	7.37 ± 0.36a
Color value of cooked EFFS			
L^∗^	57.20 ± 1.96c	46.11 ± 3.87b	42.93 ± 2.70a
a^∗^	1.70 ± 0.70a	14.35 ± 1.64b	14.06 ± 1.58b
b^∗^	13.16 ± 1.27c	8.99 ± 0.73b	7.74 ± 0.38a
Microbial analysis (log CFU/g)			
Total viable count	8.34 ± 1.12a	8.53 ± 0.84a	8.88 ± 0.85a
Lactic acid bacteria	8.33 ± 1.44a	8.32 ± 1.38a	8.88 ± 0.50a
Yeast and mold	3.64 ± 1.21a	3.29 ± 1.05a	3.51 ± 1.03a
Titratable acidity (%)	2.14 ± 0.30a	2.11 ± 0.31a	2.12 ± 0.29a
pH	4.45 ± 0.15a	4.51 ± 0.20a	4.53 ± 0.21a
Weight loss (%)	4.11 ± 1.08a	4.09 ± 1.98a	4.60 ± 1.63a
Cooking loss (%)	14.52 ± 1.88a	15.52 ± 1.32a	15.44 ± 1.90a
Texture profile analysis			
Hardness (g)	395.29 ± 40.84a	412.16 ± 33.05a	390.95 ± 48.46a
Adhesiveness (g.sec)	−5.81 ± 1.66a	−5.33 ± 1.58a	−5.47 ± 1.68a
Springiness	0.26 ± 0.06a	0.28 ± 0.04a	0.27 ± 0.05a
Cohesiveness	0.20 ± 0.04a	0.22 ± 0.08a	0.21 ± 0.66a
Chewiness (g)	21.10 ± 1.37a	22.9 ± 1.59a	23.11 ± 1.49a

Results are expressed as the mean ± standard deviation (*n* = 12). Values in the same row followed by different letters are significantly different (*p* < 0.05). RYR = red yeast rice; EFFS = Esan fermented fish sausage; L^∗^ = lightness; a^∗^ = red/green; b^∗^ = yellow/blue.

**Table 2:** Effect of the level of red yeast rice on the sensory scores and overall preference ranking of raw and cooked Esan fermented fish.

Sensory attribute	Level of RYR (% of minced fish meat)		
	0	0.35	0.70
Raw EFFS			
Color	5.45 ± 0.73a	6.65 ± 0.23b	6.46 ± 0.36b
Odor	5.73 ± 0.88a	6.21 ± 0.48a	5.77 ± 0.89a
Hand-feel texture	5.69 ± 0.47a	6.02 ± 0.41a	6.00 ± 0.35a
Overall acceptability	5.93 ± 0.20a	6.80 ± 0.32b	6.66 ± 0.13b
Overall preference ranking	2.58 ± 0.25a	1.52 ± 0.43b	1.74 ± 0.66b
Cooked EFFS			
Color	5.17 ± 0.63a	7.02 ± 0.53b	6.49 ± 0.79b
Odor	6.17 ± 0.45a	6.49 ± 0.59a	6.08 ± 0.96a
Mouthfeel texture	6.39 ± 0.6a	6.37 ± 0.78a	6.06 ± 0.94a
Flavor	6.29 ± 0.50a	6.24 ± 0.72a	6.10 ± 0.76a
Overall acceptability	5.54 ± 0.61a	6.66 ± 0.71b	6.39 ± 0.82b
Overall preference ranking	2.24 ± 0.39a	1.67 ± 0.27b	1.86 ± 0.41b

Each number is the mean ± standard deviation (*n* = 12). Values in the same row followed by different letters are significantly different (*p* < 0.05). RYR = red yeast rice; EFFS = Esan fermented fish sausage.

## Data Availability

Data are available from the corresponding author upon reasonable request.
